# Post-translational control of NLRP3 inflammasome signaling

**DOI:** 10.1016/j.jbc.2024.107386

**Published:** 2024-05-17

**Authors:** Meghan E. O’Keefe, George R. Dubyak, Derek W. Abbott

**Affiliations:** 1 Department of Pathology, Case Western Reserve University School of Medicine, Cleveland, Ohio, USA; 2 Department of Physiology and Biophysics, Case Western Reserve University School of Medicine, Cleveland, Ohio, USA

**Keywords:** cell death, inflammasome, NLRP3, post-translational modification (PTM), signaling

## Abstract

Inflammasomes serve as critical sensors for disruptions to cellular homeostasis, with inflammasome assembly leading to inflammatory caspase activation, gasdermin cleavage, and cytokine release. While the canonical pathways leading to priming, assembly, and pyroptosis are well characterized, recent work has begun to focus on the role of post-translational modifications (PTMs) in regulating inflammasome activity. A diverse array of PTMs, including phosphorylation, ubiquitination, SUMOylation, acetylation, and glycosylation, exert both activating and inhibitory influences on members of the inflammasome cascade through effects on protein-protein interactions, stability, and localization. Dysregulation of inflammasome activation is associated with a number of inflammatory diseases, and evidence is emerging that aberrant modification of inflammasome components contributes to this dysregulation. This review provides insight into PTMs within the NLRP3 inflammasome pathway and their functional consequences on the signaling cascade and highlights outstanding questions that remain regarding the complex web of signals at play.

Inflammasomes are molecular signaling complexes that assemble in response to danger and damage signals within the cell. Their formation is triggered by a wide range of stimuli, including viral, bacterial, and fungal components, and endogenous signals, such as mitochondrial damage or lysosomal disruption. Assembly of inflammasome complexes results in the activation of inflammatory caspases and cleavage of gasdermins, a family of pore-forming proteins. Cleaved gasdermins can then insert into both organelles and the plasma membrane, oligomerize, and form pores, resulting in rapid permeabilization and lytic cell death.

Inflammasome-driven pyroptosis results in the release of cytokines that serve as important mediators of the immune response. While this process is crucial for the body’s ability to fight infection and repair tissue damage, its regulation is even more crucial, as inappropriate or over-activation of inflammasomes is associated with a number of autoinflammatory diseases (1). The mechanisms underlying inflammasome activation and regulation have been the subject of intense study as the complex was first identified 2 decades ago (2). Less well studied are the signaling cascades that drive inflammasome assembly, the subsequent effect on signal transduction within the cell, and the downstream consequences of signaling cascade alteration. This review article will focus on the post-translational modifications affecting canonical (NLRP3) inflammasome signaling, inflammasome formation, and cell death as well as the downstream signaling pathways affected by inflammasome formation.

## Non-transcriptional priming

**Figure 1:**
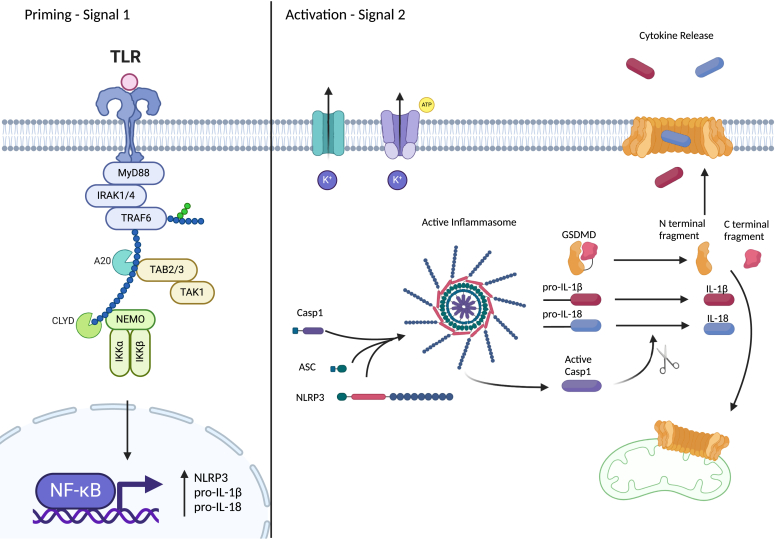
**Canonical NLRP3 inflammasome priming and activation.** The inflammasome is first primed (*left*) through toll-like receptor (TLR) signaling which drives NF-κB-dependent expression of NLRP3, pro-IL-1β, and pro-IL-18. Activation (*right*) occurs in response to a variety of pathogen and damage associated molecular patterns (PAMPs and DAMPs). This results in assembly of the inflammasome complex, activation of caspase-1, cleavage of GSDMD, and release of cytokines through the GSDMD pore.

Canonical inflammasome activation requires two independent stimuli: signal one, which primes the inflammasome, and signal two, which activates it (Fig. 1). Upon signal two stimulation, the inflammasome forms, with NACHT, LRR and PYD domains-containing protein 3 (NLRP3) associating with the adaptor protein ASC (Apoptosis-associated speck-like protein containing a CARD) and caspase-1 to form the inflammasome complex. The resulting autocleavage of caspase-1 allows for its activation and the downstream cleavage of inflammatory cytokines and gasdermin D (GSDMD). The cleaved N-terminus of GSDMD can then form pores in the plasma membrane as well as intracellular membranes, leading to cell swelling and pyroptosis, a form of lytic, inflammatory cell death.

Before signal two has an effect, the cell must be primed for inflammasome activation (signal one). Traditionally, priming was thought to occur primarily through transcriptional regulation, with Toll-like receptor (TLR) stimulation leading to increased expression of pro-IL-1β, pro-IL-18, and the NLRP3 inflammasome protein itself. However, studies have since shown that priming can also occur through post-translational or non-transcriptional mechanisms. Juliana *et al.* found that as little as 10 min of treatment with lipopolysaccharide (LPS) induces deubiquitination of NLRP3 in a process that is dependent on mitochondrial reactive oxygen species (ROS) production, leading to inflammasome activation, while ATP treatment can induce deubiquitination and activation without LPS pretreatment, in a manner that is not dependent on mitochondrial ROS (3). Further studies have shown that concurrent treatment with LPS and ATP can lead to NLRP3 inflammasome activation in a manner dependent on IL-1 receptor associated kinases (IRAK) one and four activity, although a specific phosphorylation event dependent on these kinases has not been identified (4, 5).

From a cell biological standpoint, it has been shown that upon stimulation of the inflammasome, NLRP3 is recruited to the Golgi network (6). Some studies suggest that this occurs directly through NLRP3 association with phosphatidylinositol-4-phosphate, while others propose that NLRP3 is first recruited to mitochondria-associated ER membranes (MAMs) which then localize adjacent to the Golgi (6, 7, 8, 9). The localization of NLRP3 at the Golgi is not specific to a particular inflammasome stimulus. It is hypothesized that this localization allows for the oligomerization of NLRP3, and that further phosphorylation of NLRP3 by protein kinase D allows for the recruitment of ASC and assembly of the active inflammasome, although this phosphorylation event has not yet been widely studied (6). Implicit in this cell biology and cell physiology is that post-translational regulation of each component is context and stimulation-dependent.

## NLRP3

### Phosphorylation

The central nucleator of the inflammasome, NLRP3, is subject to extensive post-translational modifications, including phosphorylation, ubiquitination, and sumoylation. The downregulation of these PTMs, *via* phosphatases or deubiquitinases also dictates signal strength. The most N-terminal PTM on NLRP3 occurs on Serine-5 and has been published as an AKT phosphorylation site (Table 1) (10, 11). AKT activity is largely dependent on extracellular signaling including insulin, IGF-1, growth factors and other cell growth/survival mediators. AKT contains a pleckstrin binding domain that anchors it to membranes, and as such, is an attractive potential modifier of NLRP3 as the cell seeks to integrate danger and growth signals (12).

**Table 1:** Phosphorylation sites within inflammasome components

Inflammasome component	Modification site	Enzyme	Activating/Inhibitory	Reference
NLRP3	Ser 5	AKT	Inhibitory	(10, 11)
	Tyr 136, Tyr 140, Tyr 143, Tyr 168	BTK	Activating	(22, 23)
	Ser 198	JNK, Pak1	Activating	(17, 18)
	Ser 295	PKA	Inhibitory	(24)
	Thr 659	Unknown	Activating	(18)
	Ser 803	Unknown	Activating	(26)
	Tyr 861	Phosphatase: PTPN22	Inhibitory	(32)
ASC	Tyr 146	Syk, Pyk2, cAbl, Jnk	Activating	(75, 76, 77, 81)
	Ser 16, Ser 193	IKKα	Inhibitory	(82)
	Tyr 60, Tyr 137	Unknown	Activating	(83)
Caspase-1	Ser 376	Pak1	Activating	(96)
GSDMD	Ser 46	AMPK	Inhibitory	(100)

Phosphorylation of Serine-5 was first identified by the Latz group using a phospho-proteomic approach (10). This residue is located at the pyrin-pyrin interaction domain and when mutated to phosphomimetic residues showed inhibition of IL-1β release. Although not studied directly, this work implied that phosphorylation of the pyrin domain inhibited homophilic interactions to inhibit inflammasome formation. In this initial identification, the kinase phosphorylating Serine-5 was not identified. Three years later, Zhao and colleagues identified AKT as the kinase mediating Serine-5 phosphorylation (11). Using an allosteric AKT inhibitor, MK2206, as well as an AKT activator, SC-79, they showed that AKT inhibition increased IL-1β release and caspase-1 activation while AKT activation decreased IL-1β release and caspase-1 activation. In contrast to a prior study which showed that MK2206 could decrease NF-κB activity and therefore Signal 1, these authors found no such effect (13). Thus, the results seem to be specific to inflammasome-activating pathways. Together these two bodies of work imply that phosphorylation can inhibit pyrin-pyrin interactions to limit inflammasome responses.

While this site of Ser five phosphorylation appears to be functional, the kinase mediating this direct phosphorylation has not been definitively identified using the criteria outlined by Cohen and Knebel (14). These criteria include demonstrating that the substrate is phosphorylated stoichiometrically, that it is phosphorylated *in vivo* in response to a known stimulus of the putative kinase, and that knockout of the kinase abrogates phosphorylation and that it can be phosphorylated directly in an *in vitro* biochemical assay (14). AKT typically phosphorylates amino acid motifs containing the sequence Arg-X-Lys/Arg-X-X-Ser/Thr-X-B where X is any of the canonical amino acids and B is a bulky hydrophobic residue (15, 16). Intriguingly, while Ser-5 on NLRP3 on the human protein contains a Lysine at the −3 position, the mouse protein does not and in fact, Serine-5 on the human protein is actually Serine-3 on the mouse protein. The mouse protein, therefore, does not have the canonical AKT phosphorylation sequence and the human protein is lacking a Lys or Arginine at the −5 position. Additionally, many protein kinases, particularly protein kinase A and protein kinase C prefer basic residues at the +3 position and these have not been ruled out as potential kinases. While there are certainly examples of imperfect phosphorylation motifs still allowing phosphorylation, it will be important to determine direct AKT phosphorylation by *in vitro* studies as well as rule out other kinases that could phosphorylate this residue.

**Figure 2:**
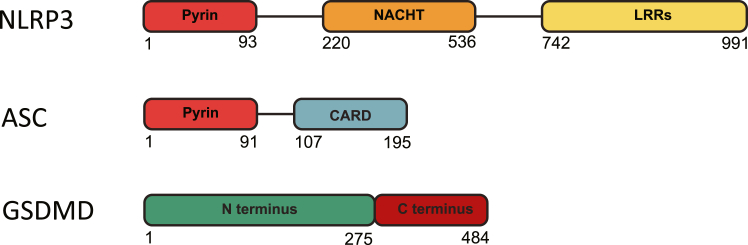
**Schematics of NLRP3 inflammasome components.** Amino acid numbers listed below each protein refer to the human protein. CARD, caspase activation and recruitment domain; LRRs, leucine-rich repeat.

Phosphorylation of NLRP3 between the pyrin and NACHT domains (Fig. 2), at Ser 198 in humans, was found necessary for NLRP3 deubiquitination and self-association, with phosphomimetic mutants inhibiting inflammasome activation in response to nigericin, a bacterial toxin derived from *Streptomyces hygroscopicus* which is known to stimulate the NLRP3 inflammasome (17). The MAP kinase JNK and the serine/threonine kinase Pak1 have both been proposed as effectors of this phosphorylation (17, 18). While this site is a potential MAPK phosphorylation site (Ser-Pro) in both human and mouse, only the mouse sequence matches the consensus Pak1 motif (Arg-X-Ser/Thr), suggesting that the responsible kinase may be species-specific (19).

Also within this linker region, Bruton’s tyrosine kinase (BTK) phosphorylation of NLRP3 promotes inflammasome activation. While most commonly associated with its role in B cell receptor signaling, BTK is expressed in a variety of immune cells, including monocytes and granulocytes. Previous studies have shown that BTK interacts with members of TLR signaling pathways, including MyD88, Mal, and IRAK-1, downstream of TLR activation, and that LPS-induced production of TNF and IL-1β is dependent on BTK (20, 21). While this would suggest that BTK is involved in the priming of the inflammasome, Ito *et al.* demonstrated that BTK colocalizes with NLRP3 and ASC upon inflammasome stimulation with nigericin and proposed that it promotes ASC oligomerization, although a specific phosphorylation site was not identified (22). In 2021, Bittner *et al.* identified four tyrosine residues (Tyr 136, 140, 143, and 168) whose phosphorylation by BTK promotes the shift of NLRP3 from intact Golgi to dispersed Golgi membranes, resulting in oligomerization and downstream release of IL-1β (23). They hypothesize that this phosphorylation alters the charge within the polybasic region of the linker, altering its ability to interact with phospholipids within the Golgi membranes. Implicit in this finding is that phosphorylation may help localize NLRP3 for oligomerization.

In the NACHT domain of NLRP3, phosphorylation at human Ser 295 by protein kinase A (PKA) inhibits inflammasome activation (24). Interestingly, PKA’s activity is dependent on cyclic AMP (cAMP), which had previously been proposed to bind to and inhibit NLRP3 (25). This more recent finding by Mortimer *et al.* raises the question of whether cAMP interacts directly with NLRP3 or if it perhaps exerts its inhibitory effect by promoting phosphorylation by PKA. Variants in residues surrounding this site have also been linked to cryopyrin-associated periodic syndrome (CAPS), a group of autoinflammatory diseases characterized by IL-1β-mediated inflammation, and S295A mutation phenocopied these variants *in vitro* (24), suggesting that this phosphorylation event could be crucial for NLRP3 inhibition.

Within the leucine-rich repeats-containing (LRR) domain of NLRP3, phosphorylation at Ser 803 in mice was found to occur upon LPS stimulation and to be necessary for NLRP3 interaction with never in mitosis A (NIMA)-related kinase 7 (NEK7) (26). Threonine 659 phosphorylation in humans was also shown to be necessary for NEK7 association (18). Both the Beutler and the Nuñez groups identified NEK7, itself a kinase, as being critical for NLRP3 inflammasome assembly, although its kinase activity was found to be dispensable in this context (27, 28). While further studies have elucidated the structural basis of the NLRP3-NEK7 interaction and suggest that it contributes to NLRP3 oligomerization, the exact function of NEK7 in this role remains unclear (29).

Acting in opposition to kinases, phosphatases can also play a key role in regulating inflammasome activity. While the tyrosine phosphatase PTPN22 is generally known for its role as a negative regulator of T-cell receptor signaling, it is also expressed in innate immune cells (30). Polymorphisms in PTPN22 have been associated with a number of autoimmune disorders, including rheumatoid arthritis and systemic lupus erythematosus (30). Prior studies have also found that PTPN22 regulates type 1 interferon production in response to TLR stimulation in myeloid cells (31). In 2016, Spalinger *et al.* found that Tyr 861 is phosphorylated upon LPS stimulation, although a responsible kinase was not identified, and that dephosphorylation by PTPN22 is necessary for inflammasome activation (32). This function of PTPN22 was specific to the NLRP3 inflammasome and loss of function resulted in a decrease in IL-1β release but not IL-6 or lactate dehydrogenase (LDH) release. Loss of PTPN22 was found to enhance colitis in mice, while a gain of function PTPN22 variant was associated with increased IL-1β in Crohn’s disease patients (32). Interestingly, a mutation at Tyr 861 (Tyr -> Cys) in NLRP3 has been reported as causative for neonatal-onset multisystem inflammatory disease (NOMID), a form of CAPS (33), further supporting the hypothesis that phosphorylation at this site is necessary for restraint of inflammasome activation.

### Ubiquitination and SUMOylation

**Figure 3:**
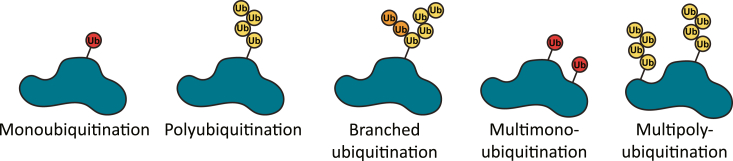
**Patterns of ubiquitination.** Ubiquitin may be added to proteins as a single molecule (monoubiquitination) or as a chain of molecules (polyubiquitination). Chains may contain a single linkage type or feature multiple, branching linkage types. A single protein may experience multiple instances of ubiquitination (multimono- and multipolyubiquitination).

In addition to phosphorylation, NLRP3 is also modified by ubiquitination and SUMOylation (Table 2). Ubiquitin is a small protein that can be attached to lysines post-translationally by E3 ubiquitin ligases. Ubiquitin can be attached as a single molecule (monoubiquitination), as chains containing one or more linkage patterns (polyubiquitination), or as branched chains (Fig. 3). One protein may contain multiple ubiquitin attachments, known as multimono- or multipolyubiquitination. The linkage patterns of polyubiquitin chains can dictate their function, with Lys (K) 48-linked and linear (M1) ubiquitination targeting a protein for degradation while Lys (K) 63-linked ubiquitination often serves as a scaffold to facilitate protein-protein interactions (34). Small ubiquitin-like molecule (SUMO) family proteins can also be attached to lysine post-translationally but do not typically target proteins for degradation. Instead, SUMOylation affects protein localization, transcription factor activity, and DNA damage repair (35).

**Table 2:** Ubiquitination and SUMOylation sites within inflammasome components

Inflammasome component	Attachment site	Linkage	Enzyme	Activating/Inhibitory	Reference
NLRP3	Lys 496	K-48 ubiquitin chain	TRIM31, Cbl-b	Inhibitory	(11, 36, 38)
	Lys 689	K-63 ubiquitin chain	Cullin1	Inhibitory	(42)
	Unknown	K-63 ubiquitin chain	Pellino-2	Activating	(45, 46)
	Pyrin domain	K-27 ubiquitin chain	HUWE1	Activating	(51)
	Lys 204	SUMO	Ligase: SUMO1, Peptidase: SENP3	Activating	(54)
	Lys 689	SUMO	Ligase: MAPL, Peptidases: SENP6, SENP7	Inhibitory	(53)
ASC	Lys 21, Lys 22, Lys 26, Lys 55	K-48 ubiquitin chain	Mul1	Inhibitory	(93)
	Lys 55	K-63 ubiquitin chain	Pellino-1	Activating	(84)
	Lys 174	K-63 ubiquitin chain	Ligase: TRAF3, Deubiquitinase: USP3	Activating	(85, 94)
	Unknown	M-1 (linear) ubiquitin chains	LUBAC	Activating	(92)
IL-1β	Lys 133	K-63 ubiquitin chain	Deubiquitinase: A20	Activating	(111, 112, 113)

Several studies have shown that ubiquitination at Lys 496 within the LRRs results in the degradation of NLRP3 (11, 36). The E3 ligases TRIM31 and Cbl-b have both been implicated in the ubiquitination of this site. TRIM31, a RING-domain-containing ubiquitin ligase, plays a crucial role in the innate immune response to viral infections, including hepatitis B, vesicular stomatitis virus, and SARS-CoV-2 (37). Song *et al.* determined that TRIM31 interacts directly with NLRP3 to promote its ubiquitination and degradation (38). Interestingly, they also found that loss of TRIM31 exacerbated alum-induced peritonitis but improved DSS-induced colitis, both of which are thought to be mediated at least in part by NLRP3. This suggests that TRIM31’s regulation of NLRP3 levels may be context-dependent. Further study of TRIM31 revealed that its ubiquitination of NLRP3 is dependent on Ser five phosphorylation, adding an additional layer of regulation by post-translational modifications, and suggesting that this ubiquitination event may specifically target active NLRP3 (11). In 2020, Tang *et al.* reported that Cbl-b, another RING-domain-containing E3 ligase, is responsible for ubiquitinating NLRP3 at this same site and that Cbl-b first requires K-63 linked ubiquitination within the LRR by RNF125 as a scaffold for its association (36). It is possible that multiple E3 ligases can ubiquitinate this site, suggesting its importance in regulating inflammasome activity, or it is possible that one of these ligases may act upstream of or in concert with the other.

A recent study of very-early-onset inflammatory bowel disease (VEO-IBD) patients identified an NLRP3 variant (R779C) in three patients which resulted in increased inflammasome activation and cytokine release (39). Further investigation revealed that this mutation resulted in markedly decreased ubiquitination of NLRP3 and increased interaction with BRCC3 and JOSD2, two deubiquitinases that are highly expressed in the colon. The authors hypothesize that the R779C variant lowers the threshold for NLRP3 activation by maintaining a low ubiquitination state, ultimately contributing to the severe inflammatory bowel disease seen in these patients (39).

One unexpected, but potentially intriguing post-translational modification of NLRP3 relates to a cell cycle E3 ligase complex. While many E3 ligases consist of a single protein, many function as complexes, including the Skp1-Cullin1-F-box (SCF) containing complex (40). In this E3 ligase, cullin1 serves as a scaffold for the assembly of the complex, Skp1 (S-phase kinase-associated protein 1) serves as an adaptor, and the specificity of the complex for a particular substrate is defined by the binding of one of over 70 unique F-box proteins (41). Thus, it exists as a trimer that mediates scaffolding and substrate recognition. Into this complex, RING-box protein 1 (RBX1) is recruited, providing a binding site for the E2 ubiquitin-conjugating enzyme. The SCF complex and the related anaphase-promoting complex (APC/C) E3 ligase are most well known for their roles in regulating the cell cycle, including the G1-S and G2-M transitions. During the G1-S transition, SCF ubiquitinates inhibitors of cyclin-dependent kinases (CDKs), promoting their degradation and allowing CDK activity, while in the G2-M transition, it targets CDK2’s necessary binding partner, cyclin E, thus blocking CDK activity (41). APC/C ubiquitinates securin, an inhibitor of sister chromatid separation.

Given their roles in this context and their roles in cell cycle progression, mitotic function and sister chromatin separation, it is somewhat surprising that components of both SCF and APC/C have been found to interact with NLRP3. Cullin1 was first identified to interact with NLRP3, and although this interaction is independent of the other SCF components (Skp1 and RBX1), it was found to repress NLRP3 activation through K63-linked ubiquitination at Lys 689 (42). Further work from this same group showed that the APC10 subunit of APC/C also interacts with NLRP3, in this case promoting inflammasome activation, although this interaction did not appear to induce ubiquitination (43). The authors provocatively hypothesize that the APC10-NLRP3 interaction promotes assembly of the inflammasome specifically during interphase, preventing activation during mitosis.

A more recent study carried this work further by demonstrating that the adaptor protein SERTAD1 can inhibit Cullin1-mediated ubiquitination of NLRP3 by disrupting the protein-protein interaction, resulting in increased inflammasome activation (44). *In vitro*, SERTAD1 knockout was associated with decreased IL-1β and IL-18 secretion, while *in vivo* loss of SERTAD1 attenuated peritonitis and experimental autoimmune encephalomyelitis (EAE) (44). Given the similarities between the SCF and APC/C complexes, it is surprising that their components induce opposite effects on the inflammasome. Further study of these interactions could answer a number of interesting questions, including how APC10 exerts its effect if not through ubiquitination and whether SERTAD1 also plays a role in regulating that interaction. Left unanswered is the role of the inflammasome in cell cycle progression or whether cell cycle status affects the threshold for activation of inflammasomes.

Ubiquitination of NLRP3 can also promote its activation, and several E3 ligases are important in the priming and assembly of the inflammasome. The ubiquitin ligase Pellino-2 has been found to promote inflammasome activation and ASC oligomerization (45, 46). The Pellino family of E3 ligases plays a critical role in TLR signaling, NF-κB activation, and upregulation of inflammatory cytokines, but Humphries *et al.* were the first to identify Pellino-2’s role in regulating NLRP3 in 2018 (46, 47). They found that Pellino-2 promotes K63-linked ubiquitination during the priming phase but does not interact directly with NLRP3. This work is supported by prior literature showing the Pellino family’s role in TLR and NOD signaling (48). Further work by Cristea *et al.* in 2021 showed that Pellino-2 colocalizes with NLRP3 in response to LPS treatment and K^+^ efflux but did not identify a direct interaction between the two proteins (45). Interestingly, a mutation in Pellino-2 associated with ocular pterygium–digital keloid dysplasia, a rare hereditary disease causing vision impairment and keloids, results in constitutive NLRP3 activation (49). Studies using patient-derived fibroblasts or overexpression of this variant found that it does not affect Pellino-2’s stability, localization, or interaction with known binding partners, leaving the mechanism of its impact, if any, on NLRP3 unknown (49).

Several other E3 ligases have been shown to directly ubiquitinate NLRP3, including HUWE1. HUWE1, a HECT domain-containing E3 ligase, plays a critical role in maintaining cellular homeostasis, and its dysregulation has been associated with tumorigenesis and metastasis in numerous types of cancer (50). Given its role in responding to cellular stress and damage, it is unsurprising to find that HUWE1 also ubiquitinates the NLRP3, NLRC4, and AIM2 inflammasomes. HUWE1 associates with the NACHT or HIN domain of the inflammasome before attaching K27-linked ubiquitin chains within the pyrin domain (in NLRP3 and AIM2) or the CAR domain (in NLRC4) (51). Loss or inhibition of HUWE1 impaired inflammasome activation, and HUWE1-deficient mice showed increased susceptibility to Gram-negative bacterial infections (51). While K27 ubiquitin linkages can promote protein-protein interactions or autophagic degradation, it remains unknown what specific function this ubiquitination plays in NLRP3 inflammasome activation (52).

In addition to ubiquitination, NLRP3 has been found to be SUMOylated at baseline on Lys 689 by the SUMO E3 ligase, MAPL. This SUMOylation is thought to suppress inflammasome activation, with deSUMOylation occurring by SENP6 and SENP7 upon nigericin stimulation (53). Conversely, SUMOylation at Lys 204 by the E3 ligase SUMO1 promotes inflammasome assembly, while deSUMOylation at this site by SENP3 attenuates activation (54). The interplay of these ligases and proteases underscores the critical importance of strict regulation of NLRP3 activity to maintain an appropriate inflammatory response.

Several other E3 ligases, including TRIM65, ARIH2, and MARCHF7, have also been found to inhibit NLRP3 activity, although specific ubiquitination sites have not been identified (55, 56, 57). Given the fact that ubiquitin ligases make up an estimated 5% of all human genes, it is unsurprising that many interactions are remaining whose mechanisms remain unknown (58). Future work in this area will likely shed increased light on the way in which inflammasome activity is regulated and how this regulation occurs in concert with other cellular functions.

### Palmitoylation

Several recent studies have also identified palmitoylation as a critical regulator of NLRP3 activity (Table 3). Through the attachment of a long-chain fatty acid derived from palmitic acid to a cysteine (S-palmitoylation) or to a serine or threonine (O-palmitoylation), palmitoylation frequently affects a protein’s ability to localize to both the plasma membrane and the membranes of organelles. In the case of NLRP3, however, it appears that palmitoylation is not involved in regulating its intracellular localization, but rather its stability and protein-protein interactions. Wang *et al.* found that palmitoylation of human Cys 844 by the palmitoyltransferase zDHHC12 promoted degradation of NLRP3 *via* the chaperone-mediated autophagy pathway, inhibiting inflammasome activation (59). Further, they demonstrate that zDHHC12 is necessary for preventing sustained inflammation *in vivo* in response to alum or LPS. Conversely, Zheng *et al.* found that palmitoylation of human Cys 837 and 838 by zDHHC5 did not affect its cellular localization or stability, but did promote inflammasome activation by enhancing NLRP3’s ability to bind NEK7 (60). These opposing consequences of palmitoylation further underscore the complex coordination of specific post-translational modifications that is necessary to regulate inflammasome activity.

**Table 3:** Other modifications of inflammasome components

Inflammasome component	Modification	Site	Enzyme	Activating/Inhibitory	Reference
NLRP3	Palmitoylation	Cys 844	zDHHC12	Inhibitory	(59)
	Palmitoylation	Cys 837, Cys 838	zDHHC5	Activating	(60)
	ADP-Ribosylation	Unknown	PARP1, bacterial ADPRTs	Activating	(69, 70, 71)
Caspase-1	S-nitrosylation	Cys 285	iNOS	Inhibitory	(98, 99)
GSDMD	Succination	Cys 192	Unknown	Inhibitory	(102)
	Palmitoylation	Cys 191	zDHHC5, zDHHC9	Activating	(105, 106)
	Oxidization	Cys 38, Cys 56, Cys 268, Cys 467	Unknown	Activating	(107)
	Oxidization	Cys 192	Unknown	Activating	(108)
IL-1β	S-glutothiolynation	Cys 1888	Grx1	Activating	(110)

### Other modifications

While the attachment of large modifications like ubiquitination and palmitoylation can have an understandably large impact on protein function, so too can the attachment of much smaller modifications, such as acetylation or nitrosylation. Several groups have found that NLRP3 undergoes S-nitrosylation in response to interferon or LPS exposure, resulting in inhibition of inflammasome assembly and activation (61, 62). However, other work has suggested that NLRP3 is not directly impacted by increased nitric oxide synthase and that instead inflammasome activity is suppressed by the effect of nitric oxide on mitochondrial homeostasis (63). It has also been shown that acetylation of NLRP3 is necessary for inflammasome assembly, while deacetylation by sirtuin two or pharmacological inhibition of acetyltransferases both result in decreased inflammasome activation (64, 65, 66). With acetylation being the most common covalent modification of eukaryotic proteins, it is unsurprising that the inflammasome components are modulated by this attachment; however, further studies should investigate the mechanism through which this modulation occurs, as this remains unknown (67).

NLRP3 is also modified through the attachment of ADP-ribose by both endogenous poly(ADP-ribose)polymerases (PARPs) and bacterial ADP-ribosyl transferases (ADPRTs). While PARPs were originally identified for their role in DNA damage repair, we’ve since come to realize that their function is much broader and more complex, and includes the regulation of enzyme functions and gene expression, as well as cross-talk with other post-translational modification mechanisms (68). ADP-ribosylation of NLRP3 was first identified to be the result of *Mycoplasma pneumoniae* infection, which produces the Community-Acquired Respiratory Distress Syndrome (CARDS) toxin (69). The ADPRT activity of CARDS was necessary for the activation of the inflammasome by the toxin, with truncated or inactive toxins producing significantly decreased levels of active caspase-1 and mature IL-1β (69). Further work has shown that in neutrophils infected with *Pseudomonas aeruginosa*, NLRP3 undergoes ADP-ribosylation catalyzed by the bacteria enzyme ExoS, which functions as an ADPRT (70). *In vivo*, response to *P. aeruginosa* infection in the cornea was regulated by the NLRP3 inflammasome (70). Work examining the role of endogenous PARP in inflammasome activation found that PARP1 knockout decreased caspase-1 cleavage and IL-1β release in response to NLRP3 inflammasome stimuli, while treatment with a PARP1 activator had the opposite effect (71). The authors hypothesize that PARP1 facilitates binding of thioredoxin-interacting protein (TXNIP) to NLRP3 to drive its activation, although they do not show this interaction directly. Further study is needed to identify sites of ADP-ribosylation within NLRP3 and other inflammasome components, and to determine its function in this context.

## ASC

In addition to inflammasomes themselves, the adaptor protein ASC (Apoptosis-associated speck-like protein containing a CARD) also undergoes post-translational modifications which can affect downstream signaling and pyroptosis. Upon inflammasome stimulation, ASC is recruited to the complex *via* pyrin domain (PYD) interactions. It then assembles into long, helical filaments which condense into a large supramolecular structure known as a “speck” (72, 73, 74). This assembly creates a large number of sites for CARD-CARD interactions and cleavage of procaspase-1, driving signal amplification. Phosphorylation and ubiquitination of ASC play crucial roles in regulating ASC speck formation.

### Phosphorylation

Phosphorylation of ASC is involved in both positive and negative regulation of speck formation. In the CAR domain, phosphorylation at human Tyr 146 is necessary for ASC oligomerization downstream of NLRP3 inflammasome activation, and resultant caspase-1 activation and IL-1β release (75). Multiple kinases have been identified as being responsible for this phosphorylation, including Syk, Pyk2, and cAbl (75, 76, 77). While tyrosine kinases typically have less stringent substrate motif preferences than other kinases, this site does not align with previously published cAbl or Syk motifs, raising questions about the likelihood of a direct phosphorylation interaction (78, 79, 80). Phosphorylation of the homologous Tyr 144 in mice also requires Syk or Jnk kinase activity, although a direct interaction has also not been demonstrated (81). Syk and Jnk were also shown to be necessary for inflammasome activation *in vivo*, with knockout mice showing a reduced response to monosodium urate (MSU) or alum-induced peritonitis (81).

ASC is also phosphorylated by IKKα in both the PYD (mouse Ser 16) and the CARD (mouse Ser 193); however, this phosphorylation restricts inflammasome activation. IKKα was found to form a complex with ASC in the perinuclear area, until inflammasome stimulation occurs, resulting in recruitment of the phosphatase PP2A which inactivates IKKα and allows ASC to dissociate (82). *In vivo*, chimeric mice expressing kinase-dead IKKα in the bone marrow compartment showed increased inflammation in response to MSU and silica (82). Further work found that treatment with a pharmacological inhibitor of protein tyrosine phosphatases (PTPases) inhibited ASC oligomerization, suggesting that dephosphorylation of tyrosine residues is necessary for speck formation (83). The same study also found that phosphorylation of human Tyr 60 and 137 was necessary for ASC function (83).

### Ubiquitination

Ubiquitination of ASC is also crucial for the regulation of inflammasome activity. The E3 ligase Pellino-1 is responsible for K-63 linked ubiquitination of ASC in the PYD, at Lys 55. This ubiquitination promotes ASC interaction with NLRP3 and oligomerization (84). Pellino-1 knockout mice were resistant to alum-induced peritonitis, with increased survival and decreased serum IL-1β. As previously stated, Pellino-2 is thought to indirectly promote the ubiquitination of NLRP3, leading to ASC oligomerization. Further work could investigate whether Pellino-2 may be acting on ASC and what, if any, redundancy exists among Pellino family members in this context. It is also important to understand the Pellino family members’ roles in priming *versus* inflammasome triggering, as this may provide valuable insight into their regulation of pyroptosis.

The TNF receptor-associated factor (TRAF) family of E3 ligases controls signal transduction downstream of pattern recognition receptors, ubiquitinating proteins that often serve as docking sites for complex assembly and signal transduction. TRAF3, in particular, is activated downstream of TLR or RIG-I engagement, as in the context of bacterial or viral infection. It is therefore unsurprising that TRAF3 also appears to play a role in promoting inflammasome activation. Within the CARD of ASC, TRAF3 drives K63-linked ubiquitination at human Lys 174, which prolongs ASC stability and promotes its aggregation, supporting inflammasome activation (85). This ubiquitination by TRAF3 is required for speck formation in response to vesicular stomatitis virus (VSV) and SARS-CoV (85, 86).

While canonically, ubiquitin chains form *via* lysine linkage to the C-terminus of another ubiquitin moiety, linear ubiquitination - where rather than a lysine linkage, the linkage occurs at the translation-initiating methionine - has been increasingly recognized as a key regulator in innate immune signaling. Within the genome, there appears to exist a single linear ubiquitin ligase complex, consisting of the proteins HOIL-1, HOIP and SHARPIN that forms linear ubiquitin chains to serve as docking sites on the target protein for signaling scaffolds (87). The importance of this linear E3 ligase complex is highlighted by the fact that SHARPIN mutations in mice cause an autoinflammatory disorder highlighted by inflammation of many organ systems, including the gastrointestinal tract and skin (88). Likewise, mutations in human HOIP cause an inflammatory disease characterized by multiorgan inflammation and immunodeficiency and mutations in human HOIL-1 cause similar phenotypes (89, 90). To date, there is a single known linear ubiquitin-specific deubiquitinase called OTULIN, which itself contains human mutations that also give rise to inflammatory disease (91). The fact that both the enzymes that catalyze the formation of linear ubiquitin chains and the enzymes that reverse these chains give rise to multi-organ inflammatory disease and immunodeficiency highlights the delicate balance that the innate immune system must strike. Too much signaling causes disease, while too little does the same. It is not surprising, then, that ASC is itself linearly ubiquitinated. Without ASC linear ubiquitination, the NLRP3 inflammasome fails to properly seed and IL-1β release is blunted (92). HOIL-1-null cells fail to properly respond to inflammasome stimulation and IL-1β responses are blunted (92). One important caveat to these studies, however, lies in the fact that inflammasome triggering requires two signals; the first to initiate NF-κB signaling to properly express NLRP3, caspase-1, caspase-11, IL-1β and IL-11, and the second to nucleate the inflammasome. Linear ubiquitination is required for both events, so it is difficult to separate an inability to prime in linear ubiquitin chain assembly complex (LUBAC)-deficient cells from an inability to trigger inflammasome nucleation. Future studies will need to address this dichotomy.

Not only does ubiquitination activate the inflammasome, but inflammasome activation can also be suppressed by the attachment of K48-linked ubiquitin chains to ASC. The E3 ligase Mul1 was found to ubiquitinate mouse lysines 21, 22, 26, and 55, resulting in decreased caspase-1 activation and IL-1β release and increased proteasomal degradation of ASC (93).

Finally, the removal of ubiquitin *via* deubiquitinase activity also regulates inflammasome activation. Ubiquitin specific peptidase (USP) three removes K48-linked ubiquitination from ASC at Lys 174, promoting ASC stability. Loss of USP3 decreases speck formation, while overexpression promotes inflammasome activation *in vivo* (94). Lee *et al.* also showed that removal of K63-linked ubiquitin from ASC by USP50 is necessary for inflammasome activation, although the mechanism is unknown (95). This would suggest that baseline ubiquitination of ASC restricts inappropriate inflammasome activation, potentially by inhibiting speck formation.

## Caspase-1

Upon activation of the NLRP3 inflammasome, caspase-1 undergoes autocleavage resulting in its activation and downstream cleavage of inflammatory cytokines and GSDMD. As such, it represents an additional node at which the pyroptotic pathway can be regulated by posttranslational modifications. To date, only one putative phosphorylation site has been identified on caspase-1 in low-throughput studies. In 2005, Basak *et al.* determined that the kinase Pak1 is activated in THP-1 monocytes upon stimulation with LPS (96). They further demonstrated that caspase-1 cleavage is diminished in the presence of kinase-dead Pak1 or with mutation of caspase-1 at human Ser 376, the site of a potential Pak1 phosphorylation motif. They demonstrated phosphorylation of caspase-1 using an *in vitro* kinase assay, but to date, *in vivo* phosphorylation of Ser 376 has not been formally demonstrated (96). Given the more recent work described above suggesting that Pak1 is responsible for NLRP3 phosphorylation, it is apparent that further study is needed to elucidate its specific interactions with inflammasome pathway members.

The Lamkanfi lab has demonstrated that caspase-1 is ubiquitinated in response to multiple inflammasome stimuli, including ATP and nigericin (97). This ubiquitination was abrogated upon treatment with Ac-YVAD-cmk, a caspase-1 inhibitor, suggesting that the modification requires autocatalytic caspase-1 activity, although whether the activity is required for modification in *cis* or *trans* is not known. The authors speculate that this ubiquitination may serve as a negative feedback mechanism to inhibit inflammasome activation, but a specific site or ubiquitin linkage pattern was not identified, leaving its function unknown (97).

Several studies have also shown that caspase-1 is S-nitrosylated at its catalytic site, human Cys 285 (98, 99). This nitrosylation inhibits caspase-1 activity, resulting in reduced cleavage and release of IL-1β both *in vitro* and *in vivo*. Mice lacking the enzyme inducible NO synthase (iNOS) had significantly higher plasma levels of IL-1β upon LPS stimulation compared to wildtype mice (99). The effect of NO treatment was incompletely reversed by treatment with dithiothreitol (DTT), suggesting that nitric oxide may play other roles in modulating inflammasome activity, in addition to modifying other members of the pyroptotic pathway. As will be highlighted below, however, it will be important to understand the stoichiometry of this nitrosylation event as a low stoichiometry event may have more local effects than a higher stoichiometric event.

## Gasdermin D

Inflammasome assembly results in the activation of caspase-1, which proceeds to cleave GSDMD. Once cleaved, GSDMD forms pores in the plasma membrane, resulting in swelling and rupture of the cell. While inflammasomes were first identified in 2002, it was only in 2015 that GSDMD was identified as the executioner of pyroptotic cell death, and as a result, relatively little is known about the effects of post-translational modifications on its function (2). However, several recent studies have made progress in identifying key modifications.

While there are several predicted phosphorylation sites within GSDMD, thus far only one site has been identified experimentally. Chu *et al.* found that AMP-activated protein kinase (AMPK) phosphorylates Ser 46 in the cleaved N terminal fragment of GSDMD preferentially over the full length protein (100). This phosphorylation inhibited oligomerization and pore formation, acting as a potential negative regulator of pyroptosis. In an *in vivo* tumor model, phosphomimetic mutation of this site (S46D) significantly reduced the ability of GSDMD’s N terminus to mediate pyroptosis and anti-tumor immunity (100).

Recent work from our lab utilized the PhosphoSitePlus database of PTMs along with databases of human SNPs to identify four potential polymorphisms at three putative sites of post-translational modification within GSDMD (S181L, K204T, T251M, T251R) (101). Mutation at these sites could potentially disrupt phosphorylation or ubiquitination; however, we demonstrated that there was no effect on GSDMD autoinhibition, pore formation, or cell death. This was in contrast to SNPs predicted to disrupt key structural sites, which inhibited pyroptotic cell death or greatly attenuated IL-1β release.

GSDMD is also modified by succination in response to a build-up of Krebs’ cycle intermediates in activated macrophages. Work from the Fitzgerald lab found that exposure to dimethyl fumarate (DMF) or buildup of endogenous fumarate resulted in succination at mouse Cys 192, which inhibited the ability of caspase-1 to bind and cleave GSDMD, as well as GSDMD’s ability to form pores (102). *In vivo*, mice treated with DMF showed decreased symptoms in an experimental autoimmune encephalitis (EAE) model, along with decreased accumulation of the N-terminal fragment of GSDMD in the CNS. In human multiple sclerosis patients taking dimethyl fumarate, levels of IL-1β and N-terminal GSDMD were reduced in peripheral blood mononuclear cells (102). This cysteine is also targeted by the pyroptosis inhibitors necrosulfonamide and disulfiram, further underscoring its importance in the assembly of the GSDMD pore (103, 104).

Several recent studies have also identified the importance of lipidation of GSDMD. Palmitoylation at human Cys 191 is necessary for the translocation of the N terminal fragment to the plasma membrane and the formation of a pore (105, 106). This lipidation is mediated by the palmitoyl acyltransferases zDHHC5 and zDHHC9 and is also dependent on ROS production (105, 106). While unmodified GSDMD can associate directly with the lipids of the inner membrane leaflet, the authors of these studies posit that palmitoylation is required for pore formation and plasma membrane rupture.

The production of mitochondrial ROS is also responsible for the oxidation of residues throughout GSDMD. Wang *et al.* identified human Cys 38, 56, 268, and 467 as being oxidized upon NLRP3 inflammasome stimulation, and found that mutation of these four residues dramatically reduced the efficiency of caspase-1 cleavage of GSDMD (107). Devant *et al.* found that mouse Cys 192 is also oxidized in a ROS-dependent manner and that this oxidation enhances pyroptosis (108). Given that GSDMD is also thought to be succinated and palmitoylated at this same site, many questions remain. What determines the priority or sequence of modifications at this site? Are all three modifications required in parallel, across copies of the protein? How is their interplay controlled? The multiple modifications at this key cysteine underscore the importance of crosstalk between post-translational modifications and the effects that this can have on protein function.

## IL-1β

Pro-Il-1β is cleaved by caspase-1 after inflammasome activation, and the processed form is released through the newly formed GSDMD pores into the extracellular space. Once released, IL-1β induces fever and stimulates inflammation, thereby playing a key role in the ability of pyroptotic cell death to invoke an immune response (109). Although not as extensively modified as other members of the inflammasome pathway, IL-1β′s activity is also regulated by post-translational modifications.

Work by the Luo lab has shown that IL-1β is S-glutothionylated at Cys 188, a highly evolutionarily conserved residue (110). This glutothionylation protects IL-1β from inactivation due to irreversible ROS-induced oxidation at this cysteine. The thiol transferase Grx1 is responsible for deglutothiolynation of IL-1β, which the authors hypothesize contributes to the maintenance of homeostasis in times of cellular stress (110).

Multiple studies have also demonstrated that Il-1β is ubiquitinated (111, 112, 113). Duong *et al.* found that pro-IL-1β is specifically K63-ubiquitinated at Lys 133 and that this ubiquitination contributes to the processing of IL-1β, as both cleavage and release of mature IL-1β were reduced with K133R mutation (112). This study also identified the deubiquitinase A20 as being responsible for the deubiquitination of IL-1β at this site (112). In contrast to this study, which found that ubiquitination does not affect the stability of pro-IL-1β, more recent work found that ubiquitination at Lys 133 promoted proteasomal degradation, and loss of ubiquitination resulted in increased stability and bioactivity of IL-1β (111).

This evidence suggests that the consequences of inflammasome activation are regulated not only within the components themselves but also at the level of downstream messengers, which can in turn have broadly disseminated physiologic impact.

## Outstanding questions

We have catalogued a list of post-translational modifications that have been shown to affect NLRP3 inflammasome nucleation, activation, deactivation and signal response. Implicit in this extensive list is the fact that these modifications must in some way integrate to generate a specific signal on a whole cell basis. Do these modifications occur independently or in concert? Does one PTM dominate over another? Do signaling pathways cooperate to regulate inflammasome function? What is the stoichiometry of these post-translational modifications? Importantly, what happens to active signal transduction pathways when the NLRP3 inflammasome is triggered? Remarkably, while much effort has been spent mapping the PTMs, very little is known about their integration.

### Do these modifications occur independently or in concert?

Signal transduction cascades within a cell broadly integrate a cellular response to a stimulus. The case of inflammasome formation is complicated by the fact that it requires two signals - typically a TLR agonist like LPS to allow expression of NLRP3, caspase-1, caspase-11 (in mice), IL-1β and IL-18, and a second signal to trigger the inflammasome to nucleate. In the case of the priming signal, multiple signal transduction pathways are activated including NF-κB, p38, JNK, IFN, and ERK. Additionally, over the course of exposure to signal one, autocrine and paracrine effects may take place. For instance, LPS exposure causes TNF release, which can then signal back to the same cell or neighboring cells to initiate a TNF response (114). Thus, when signal two initiates inflammasome formation, multiple signaling pathways are already activated. Many of the pathways leading to the PTMs highlighted above may already have been active when the cell is exposed to signal two, while others may be naive when exposed to signal two. In addition to this potential timing difference of the PTMs, on a per-protein basis, it is unclear whether two PTMs from two separate pathways are present and active on the same protein or on separate proteins. This becomes important when one considers the effect on an individual inflammasome nucleation and activation response (or its potential dissolution) *versus* a cellular or population-based response. Unfortunately, at this point, we don’t have the answer to this important question.

### Does one PTM predominate over another?

Implicit in the integration of signaling *via* PTMs on inflammasome components is the question of whether individual PTMs dominate over others. Does an activating phosphorylation on ASC override a degradative ubiquitination on NLRP3 for instance? Does the timing of the formation of the PTM matter—for instance, once the inflammasome is nucleated, do the PTMs on ASC or GSDMD then predominate? Can combinations of PTMs on GSDMD alter the timing of pore generation or affect the size of the overall pore? All of these questions matter on a cellular basis, especially as one considers signal integration.

### Do signaling pathways cooperate to regulate inflammasome function?

Inflammasome nucleation and subsequent caspase-1 activation have generally been thought to be an all-or-none response, but little is known about the timing of inflammasome nucleation or subsequent caspase-1 activation. Do some signal transduction pathways alter inflammasome components to hasten or slow inflammasome nucleation and subsequent caspase-1 recruitment? Does this affect the timing and amplitude of IL-1β and IL-18 release, and how does this affect the multitude of other signaling responses, like mitochondrial dysfunction, which are known to occur when the inflammasome is activated and GSDMD is cleaved? Will disparate signals on the cell, such as those that occur with whole bacteria infection, converge on the inflammasome to generate a coordinated response, or do these signaling pathways compete with one another? How would this affect adaptive immune coupling? As we move forward into the next decade of inflammasome research, all of these questions become important.

### What is the stoichiometry of these post-translational modifications?

Stoichiometry of PTMs is crucially important. While there are examples of 100% of the protein being modified with a PTM, it is far more common for a PTM to go from extremely low stoichiometry to low stoichiometry (115). For instance, if 0.01% of a protein is modified, but that changes to 5% of the protein upon signal activation, that is a 500-fold increase. In contrast, moving from 10% of the protein being modified to 100% only causes a 10-fold increase. Given this, evolutionarily, most PTM stoichiometry falls in the former range. Consideration of the stoichiometry of the PTM then becomes paramount. In the case of degradative ubiquitination, the PTM may only target active proteins and thereby get rid of active complexes. In this case, low stoichiometry would be effective, but if the degradative ubiquitination targeted the entirety of the protein, active or not, low stoichiometry might not have the desired effect. In the case of PTMs that increase the activity of a protein, low stoichiometry can amplify a response, while in the case of PTMs that decrease activity, a high stoichiometry would be necessary. Some PTMs alter cellular localization, and in these cases, a low stoichiometry event would favor an activating location. In contrast, a high stoichiometry event would be required for inhibitory cellular localization. A classic example of this stoichiometry would be the NF-κB pathway, where IκBα undergoes phosphorylation to target it for degradation, thereby releasing the NF-κB transcription factors to enter the nucleus and perform their function (116). No phosphorylation on IκBα allows it to sequester the NF-κB transcription factors in the cytosol while even low levels of phosphorylation of IκBα can cause its degradation. This example also allows a graded response whereby little, some, or all of the NF-κB transcription factors can be released, depending on the level of IκBα phosphorylation This gradation underlies the difference in NF-κB target gene expression achieved with muramyl dipeptide (MDP)-induced NF-κB activation, which induces low IκBα phosphorylation, *versus* TNF-induced NF-κB activation, which induces high IκBα phosphorylation. Stoichiometry must impact the multitude of inflammasome protein PTMs; however, to date, this has not been studied.

### What happens to active signal transduction pathways when the NLRP3 inflammasome is triggered?

Most studies of the NLRP3 inflammasome have focused on its immediate cellular effects - cytokine release and pyroptosis - as well as its cell biological effects, such as mitochondrial dysfunction and cytoskeletal derangement. Little is known, however, about its effects on downstream signal transduction pathways. While inflammasome priming is known to induce widespread signaling effects, including the activation of the NF-κB, JNK, ERK, and p38 pathways, a major inflammatory insult that causes inflammasome activation must also affect these signaling pathways. An understanding of this becomes crucial when one considers that a subset of inflammasome-activated cells will not die and will become hyperinflammatory while others will begin to initiate other cell death pathways like the apoptotic cascade (117). In fact, our own work has shown the interconnectedness of apoptotic and pyroptotic cascades upon chemotherapy exposure in acute myeloid leukemia cells (118). Unfortunately, to date, there have been few studies that highlight the downstream signal transduction pathways affected by NLRP3 inflammasome activation outside of mitochondrial dysfunction and apoptosis. It will be important to address this in the future.

## Conclusion

Broadly speaking, post-translational modifications serve distinct functions within the cell. Firstly, they can impact protein localization on a large or small scale. This can result in the relocation of the protein to a number of subcellular compartments, including the nucleus, endoplasmic reticulum, and Golgi apparatus, but can also influence a protein’s position within a compartment, in relation to other proteins or organelles. Secondly, they can affect protein stability and degradation. While this role is most commonly associated with ubiquitination, nearly all PTMs can contribute, including phosphorylation, methylation, and acetylation. Thirdly, they can regulate enzymatic activity. This is seen in the classic example of kinase activation, where phosphorylation promotes a conformational change in the activation loop to allow substrate binding and activity. Finally, they can influence protein-protein interactions by allowing or inhibiting the association of binding partners. In this context, larger PTMs, such as ubiquitination, may serve as a scaffold for colocalization of proteins, while other PTMs may influence the charge or conformation of a protein to promote or inhibit interactions with binding partners. For some of the PTMs described in this review, a putative function has been proposed. For example, BTK phosphorylation of NLRP3 is thought to induce its translocation from intact to dispersed Golgi, and TRIM31-dependent ubiquitination of NLRP3 promotes its degradation. However, for the majority of the known PTMs of inflammasome components, the mechanism through which they exert their effects has not been characterized. If future work hopes to harness manipulation of these PTMs for therapeutic benefit, we must first fully understand their functions.

Another important consideration is the stoichiometry of these modifications and the interplay between them. As shown above, most members of the pyroptotic pathway are extensively modified at a number of different sites. Little work has demonstrated the influence that one modification might have on another in this context, whether specific combinations of PTMs can drive different outcomes, or what fraction of the total protein must be modified.

Further research is also necessary to consider the impact of inflammasome activation on other cellular processes. It would seem highly likely that other pathways are perturbed as a result of mitochondrial disruption, changing ion concentrations, and activation of inflammatory caspases. How are levels of protein expression changed following stimulation? What, if any, action is taken to oppose activation? Which pathways are prioritized by the cell in the face of impending pyroptosis? Does the cell “sacrifice” essential functions in favor of fulfilling its role as an immunologic sentry, releasing warning signals into the extracellular environment at the expense of its survival? Answering these and other questions is key to advancing our understanding of pyroptosis and its role in both health and disease.

## Conflict of interest

The authors declare that they have no conflicts of interest with the contents of this article.
